# Patient reports of the outcomes of treatment: a structured review of approaches

**DOI:** 10.1186/1477-7525-12-5

**Published:** 2014-01-14

**Authors:** Helen Lloyd, Crispin Jenkinson, Monica Hadi, Elizabeth Gibbons, Ray Fitzpatrick

**Affiliations:** 1 Health Services Research Unit, Nuffield Department of Population Health, University of Oxford, Rosemary Rue Building, Old Road Campus, Headington, Oxford, UK

**Keywords:** Patient evaluation, Hospital, Hospital care, Treatment satisfaction, Review, Transition items, Quality of care

## Abstract

Patient reports or ratings are essential for measuring the quality of patient care. Measures designed for this purpose tend to focus on the processes and structures of care rather than the outcomes of it. The latter is arguably the most valid indicator of the quality of care patients receive. Typically this information is gathered by probing patient satisfaction with treatment as part of an investigation of satisfaction with hospital care. More recently patient ratings of the outcome of treatment have been obtained to measure treatment efficacy in clinical trials. However, a more direct approach is to ask patients to assess the benefit of treatment on their current health status. We performed a structured literature review on patient reported satisfaction with outcomes of treatment and direct patient assessments of the same. The purpose of this was to identify suitable candidate questions for a short instrument to tap patient evaluations of in-patient hospital interventions. Articles were included if they dealt with patient satisfaction or patient assessment of the outcomes of treatment. Articles were excluded if they dealt more generally with patient satisfaction with care. We identified 169 papers, 79 were included in the review. The findings of this review suggest that there are a number of benefits of directly asking patients to assess the outcome of hospital treatment. Importantly this approach reflects outcomes relevant to the patient and is also more likely to reflect patient report in routine clinical practice. There is also evidence that such approaches have face validity and construct validity. The problems associated with this approach (i.e. response bias), are those common to patient reported outcome surveys, but employing appropriate strategies can minimize them. Furthermore, employing a simple set of questions that asks patients to assess the outcomes of treatment they receive can be time and resource efficient in comparison to administering lengthy measures. This approach could be tested for potential generic use as an evaluative measure for patients in hospital settings.

## Background

The importance of measuring Patient Reported Outcomes (PROs) in health is now widely accepted, and complements the data collected by clinical observation, or by the assessment of a particular pathophysiological process. In particular, patient reported outcome measures (PROMs) are essential for measuring the quality of medical or hospital care patients receive. This can be assessed in relation to structural care provider characteristics e.g. organisational setting and resources, the processes undertaken to provide care, and the outcome or change in a patient’s health status resulting from the intervention [1]. It is therefore surprising that until recently patient evaluations have focused on the structures and processes of care, rather than the outcomes of it, particularly since the latter are arguably the most valid indicators of the quality of care received [2].

Patient ratings of improvements or outcomes of treatment have commonly been obtained by eliciting patients’ perceptions as part of an investigation of satisfaction with hospital care. More recently patient ratings of the outcome of treatment have been obtained to measure treatment efficacy in clinical trials [3]. Another mechanism for investigating PROs is by directly asking patients to evaluate the impact of treatment on their current health status. This is often achieved by using a global assessment question or transition question which asks patients to compare their current health status with that of a pre-treatment time point.

Our aim was to investigate the commonly used approaches described in the literature that have been used to elicit patient reports of the outcomes of treatment, and identify the key advantages and disadvantages of their use. This was to identify suitable candidate questions for a measure to tap patient evaluations of hospital treatment. To do this we carried out a structured review of the literature and focused on articles concerned with patient satisfaction with the outcomes of treatment or direct patient assessment of the same.

## Methods

**Figure 1:**
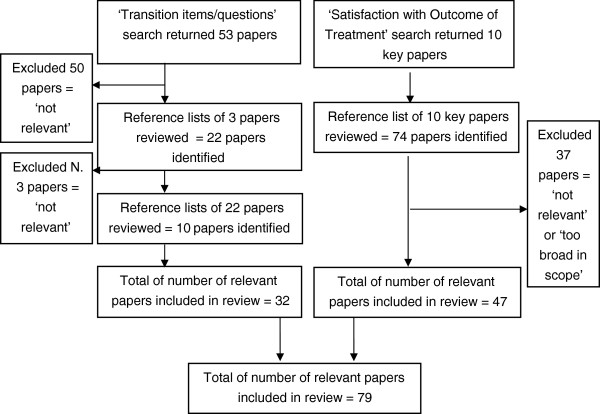
Consort diagram of review process.

We used a dual search procedure to identify articles: search (1) focused on the literature concerning patient satisfaction with the outcomes of treatment, and search (2) focused on the literature on transition questions or similar approaches. A search of Scopus and PubMed (all entries since 1970) was performed using combined terms. We also identified key articles and searched their reference lists. We screened articles detailing empirical studies, reviews, consensus statements and expert reports that either, assessed, investigated or discussed patient satisfaction with the outcomes of treatment or transition items/questions. Papers were excluded unless they described approaches to garner patient satisfaction with treatment or transition questions and related approaches (see Figure 1). We were explicitly interested in the methodological issues associated with these approaches e.g. advantages/problems (biases etc.).

### Findings

#### Article identification process

An initial scope of the PROM literature suggested that relevant papers would be found in two literature clusters; the treatment satisfaction literature and papers using or discussing transition items/external anchor questions/global judgements to validate a PROM measure. A search of the databases described above identified 53 articles relating to transition questions or related questions (i.e. global evaluative judgements). We excluded 50 papers as they contained the word ‘transition’ but did not contain a measure or questions of a transition type. From the reference lists of the 3 remaining papers 22 additional papers were produced, of these 3 were excluded for the same reasons as above. Of the remaining 22 papers 10 additional papers were identified and included, resulting in 32 papers that were included in the transition item review.

Ten key papers concerning patient satisfaction with the outcomes of treatment were identified from a review of the above databases. Scrutiny of their reference lists produced 74 papers of possible relevance, of these 37 were excluded because they dealt more broadly with the patient satisfaction. We therefore included 47 papers in this strand of the review. A total of 79 papers from both search streams were therefore the subject of the combined review.

#### Approaches to elicit patient reports on the outcomes of treatment

Our literature review confirmed that the two most commonly used approaches to elicit patient reports on the outcomes of treatments were measures of patient satisfaction and measures which employed global ratings of improvement. Other approaches exist which, while not strictly within the scope of this review deserve highlighting. One such approach is to measure the outcomes of treatment against patient specific valued goals, an example of which is the Patient Generated Index (PGI) [4]. Such measures allow for an assessment of the benefit of treatment against patient specific expectations, often employing a more complicated format with sets of linked transition questions. Such measures may be particularly good at detecting clinically significant change over time, but their complexity may render them less readily useful for some patient populations. This review therefore focused the two most commonly used approaches.

Measures of Patient Satisfaction with the outcomes of treatment are used frequently, and often as a consequence of the importance of collecting clinical trial data with an interest in treatment outcomes important to patients. A second approach is to use a Global Rating of Improvement to assess the benefits of a treatment received; this often takes the form of an overall evaluative question. Health Transition Question(s) (HTQs) are a type of global evaluation question which directly ask patients to assess whether their health or functioning has stayed the same, improved or worsened when compared with a previous (often pre intervention) time point. The latter two approaches reflect a steer away from the construct of satisfaction in recognition of problems associated with this approach e.g. positive skews in data. The following sections of this paper present a selection of findings from the literature review that serve as typical examples of these approaches. Table 1 details a selection of measures that are good examples of well validated and robust measures used to elicit patient reports of the outcomes of treatment. The latter sections of the review outline the advantages and disadvantages of using such approaches and potential strategies to consider when using these methods.

**Table 1:** A selection of well designed measures to elicit patient report of the outcomes of treatment

**Type of approach**	**Title**	**Description**	**Comments on psychometric properties**
**Satisfaction with/assessment of the outcomes of treatment**	Diabetes Treatment Satisfaction Questionnaire (DTSQ). [5]	An 8 item measure of patient satisfaction with diabetes treatment.	Developed by qualitative work to ensure comprehensive and authentic issues were covered. Assessed psychometrically and analysed in relation to covariates.
	DTSQc [6]	Revised version of the above.	Detects greater responsiveness to improvements than the original DTSQ.
	Oxford Elbow Score (OES) [7]	A 12-item PRO developed to assess the outcomes of elbow surgery.	Shown to be valid, reliable and sensitive to change after rigorous testing.
	Questionnaire on the perceptions of patients about shoulder surgery. [8]	A 12-item PRO for patients having shoulder operations.	A short, practical, reliable, valid outcome measure that is sensitive to clinically important changes.
	Questionnaire on the perceptions of patients about total hip replacement. [9]	A 12-item PRO for patients having total hip replacement (THR).	As above.
	Questionnaire on the perceptions of patients about total knee replacement. [10]	A 12-item questionnaire for patients having a total knee replacement (TKR).	As above.
**Measures containing transition items/global ratings of change**	The Evaluation Ranking Scale (ERS) [11]	The ERS asks patients to rank and then rate six dimensions or characteristics of the services they have received.	Compared with a global measure of satisfaction the ERS was more specific, more discriminating, and resulted in lower satisfaction scores [11].
	Patient Judgements of Hospital Quality (PJHQ) [12]	Designed to assess the health change associated with hospital stay/treatment over 11 scales.	This measure was subject to extensive and rigorous devolvement and testing that included patient reported open-ended responses about the quality of hospital care, and interviews with hospital administrators, physicians and nurses [12].
	Patient Global Impression of Change Scale (PGIC) [13]	Measures patient evaluations of their health change in relation to treatment.	Captures what patients consider to be important changes in pain ratings [14] and cancer specific quality of life scores [15,16]. Also a potential correlate of clinical opinion [17]. Used in trials of chronic pain [17,18] and recommended as a core outcome measure of global improvement [19].
	The Functional Status Index (FSI) [20]	A patient specific measure of change in maximal physical, mental, and emotional function with a transition component that measures change from patient specific norms.	As part of the development it was compared with the Sickness Impact Profile (SIP) [21] and performed well, showing sensitivity to change over time [22].
	The Health Transition Index (HTI) [23]	Patient rated change in health between two time periods using a 5 point ordinal scale (1 = much better than a year ago; 2 = somewhat better than a year ago; 3 = about the same; 4 = somewhat worse than a year ago; and 5 = much worse than a year ago)	HTI was used as an external anchor to assess the responsiveness of the SF36 [23], the HAQ [24] and a disease specific health status measure AIMS2 [25] in psoriatic arthritis [26] and detected as much change as clinical examination [26].
	Short Form 36 (SF36) [23]	The SF-36 is a health survey with 36 questions. It yields an 8-scale profile of functional health and well-being scores as well as psychometrically-based physical and mental health. The HTQs have five response categories from “much better” to “much worse”.	The HTQ was assessed among a large general practice sample and correlated well with change measured prospectively [27]. The discriminative properties of the HTQs were demonstrated in a similar large population study against prospective change [28]. This study was able to successfully distinguish groups whose health had improved compared to those whose health deteriorated.

#### Measures of treatment satisfaction

Within the treatment satisfaction literature we identified three methods commonly used; a single global evaluation question i.e. *“How satisfied are you with your current treatment?”* a set of separate measures for each aspect of treatment received, and a composite measure comprising of a global item and a set of separate items [29]. Treatment satisfaction measures also usually include a Likert scale with some also including a visual analogue scale (VAS).

Patient satisfaction with outcomes of treatment has been measured in a vast array of health conditions and procedures. In particular, the fields of diabetes care [5,6,30], orthopaedic surgery [8-10,31], renal treatment [32] and asthma [33] have been fruitful areas for this research, and stand out for applying methodological rigour in the development of measures to assess this (see Table 1). The acceptability of treatments has also been measured in relation to behavioural treatments for children with conduct disorders [34] and mental health treatments [35].

#### Global ratings of improvements/change and health transition questions

As a consequence of some of the problems associated with the data on satisfaction (i.e. positive data skews and undifferentiated data sets), researchers have developed other approaches to obtain this information. The main alternative is to gather patient ratings of improvement or assessments of health change in response to treatment i.e. *“Overall, how would you compare your health with the way it was before your surgery, is it much better now, a little better now, about the same, a little worse or much worse?”.*

One such approach is to collect a Global Rating of Change (GRC) related to treatment. This approach provides an opportunity for patients to combine all of the components of their experience (e.g. pain relief, improvements in functioning) into one overall evaluative measure of the treatment they receive [19]. These approaches have also been used to investigate participants’ judgments of the clinical importance of change in other outcome measures [14,36]. These questions are commonly used as an external anchor by which to assess the responsiveness of measure to patient rated meaningful health change (See the FACT (Functional Assessment of Cancer Therapy questionnaire and Health Related Quality of life in a study of oncology patients [15])). Global ratings of change have also been used to elucidate clinically important changes in scores in quality of life of instruments in chronic heart and lung disease [37,38], in asthma [38] and cancer treatment [39]. Indeed, Jaeschke et al. (1989) concluded that in the absence of a gold standard measure external global ratings represent a credible alternative for establishing the meaning of change in a new measure [37].

An example of a measure specifically designed for this purpose is the Patient Global Impression of Change Scale (PGIC) [13]. This rating scale measures patient evaluations of their health change in relation to treatment from “very much improved” to “very much worse” using a visual analogue scale, and has two variants; one for use by the clinician and one for use by the patient. The PGIC has been used in trials of chronic pain [17,18] and recommended as a core outcome measure of global improvement [19].

Central to the importance of measuring PROs are methods that assess the changes in health related status over time. Health Transition Questions (HTQs) do this by directly asking patients to assess whether they consider their health or functioning to have stayed the same, improved or worsened compared with a previous (often pre intervention) time point i.e. *“The last time we talked, you said that (physical activity from the baseline index or previous transition) was the most physically strenuous thing you could do? In terms of physical activity now are you: much better, slightly better, the same, slightly worse or much worse?”.*

HTQs are employed in a number of ways in health status measurement, and with the exception of studies that have assessed a HTQ within a measure [27,28,40-42], and the exception of several notable papers [43-45] there has been little discussion of the widespread use of these questions. This is perhaps a reflection of their perceived usefulness in the absence of a gold standard, much like global questions, they are often used as an external measure or benchmark by which to compare the responsiveness of an existing measure [15,37,46-51], or during development of a new measure [7-10,22,52,53]. Studies conducted for the latter purposes thus perform indirect assessments of HTQs in the process of using them as the external benchmark. Studies that directly assess a HTQ within a scale often do this by measuring change assessed prospectively, i.e. by calculating change scores in samples known to have experienced a clinically important change compared to those that have not experienced such a change [28]. Patient and clinician ratings of patient health change were collected to assess the responsiveness of the Sickness Impact Profile (SIP) and the American Rheumatism Association (ARA) functional scale [44]. This study found that only changes in SIP physical dimension and patient self-rating showed significant correlation with clinically estimated changes, and that transition items registered changes in clinical status that were not detected by the functional scales [54]. The benefit of using HTQs to probe patient evaluation has been acknowledged by several authors [41,53], mainly as a result of their validity and practicality. Further support for HTQs is provided by studies that assess health status measures against HTQs and standard clinical measurement [24,36,41,42,48,49].

## Discussion

### Advantages of eliciting perceptions of satisfaction with the outcomes of treatment or transition items/global ratings of improvement

The findings of this review suggest that there are a number of advantages to using the above approaches for evaluative purposes. Particularly when short measures are employed or single global questions are grouped with HTQs that probe experiences of adverse events or side effects. For example, to assess the accuracy and sensitivity of measures designed to specifically probe PROs in orthopaedic surgery, treatment satisfaction questions have been posed alongside direct HTQs as an external anchor [7,8]. This form of a HTQ is routinely used to assess the sensitivity of outcome measures in medicine [53,55] and more specifically patients with arthritis [54]. Using such a combination will help ensure that core domains cited in the literature related to treatment outcomes are covered. The main advantages are outlined below:

#### They reflect outcomes relevant to the patient

Dawson et al. [52] found that patient satisfaction with surgery poorly correlated with clinician ratings at follow up, and therefore provided more evidence of the difference between patients’ and clinicians’ perceptions of which aspect of outcome to rate as important [56,57].

#### They reflect patient reports in routine clinical practice

There is evidence that retrospective questions that probe satisfaction with outcomes of treatment (or perceptions of the same) reflect patient report in routine clinical practice and are therefore easier to incorporate into clinical decision making and care [46]. Support for this comes from a variety of studies across clinical specialties [58].

#### Face validity

The use of HTQs and treatment outcome questions as anchors to assess the responsiveness, reliability and validity of numerous disease specific [7,52] and generic measures [22,26,27,51] suggests that these questions do have an acceptable level of face validity. The development of several measures to elicit patient report of hospital stay (PJHQ & ERS) [11,12] and patient rated clinical change (PGCI & GROC) [13,37] have also been subject to content validation with patients to understand the meanings they ascribe to the questions posed.

#### Construct validity

Evidence to suggest that retrospective items of treatment outcome have construct validity has been reported from studies of hip replacement (THR) surgery [59], lower back pain [47,60], and diabetes [5,30]. Generic measures such as the PJHQ, the PGCI and the ERS also capture the perceptions of treatment success across a range of interventions and health conditions, with the majority of evidence from studies assessing the PGCI *(ibid)*.

#### Sensitive to change

Retrospective measures of change in health may be more sensitive to change than serial measures [36]. Several studies have also demonstrated that transition items register changes in clinical status that were not detected by the functional scales [54], and are better at discriminating the health status of patients than generic measures [50]. Studies that have compared measures that contain HTQs against those that do not, have found that HTQs detect clinically important change comparable to that detected by clinical endpoints [61] and better than calculated change scores in health-related quality of life [36,42]. In addition, external transition questions may be better at recognising deterioration in sub groups of patients than a generic measure [42].

#### Less demanding in time and resources

Directly asking patients to retrospectively assess the benefit of treatment or change in their health status is less demanding in resources than collecting prospective data [6,62]. For example, a meta-analysis of 150 pain treatment studies concluded that a global measure of treatment efficacy provides similar data to that derived from hourly measurements, and that the pooling of data permitted the determination of the absolute and relative efficacy of pain treatment for a substantial number of patients (e.g. 1000 or more) [63]. Compared with the cost and labour implications of using traditional prospective measures this method could contribute a substantial cost and labour saving for the NHS.

### Problems with these methods

There are however a number of problems with using these methods. Global evaluation questions tend to be crude and not accurately reflect satisfaction (which is often an amalgamation of several topics) thus masking differentials. Conversely, lengthy questionnaires can be burdensome for routine treatment evaluations. The most notable problems associated with using the above approaches are:

#### Non-response bias

A review of patient evaluations of hospital care reported similar factors to those mentioned in the literature that influence response rates in satisfaction surveys [64]. For example, non-response bias was found to positively skew results, and while little is known about why people fail to respond to surveys, there is some evidence that it is related to illness severity (ibid).

#### Acquiescent response bias

Positive skews in data sets may also be a consequence of acquiescent responses, which are common problems with PROMs [3]. However, patient response in surveys that focus on satisfaction with treatment may be a greater risk of these biases since as a construct it is intrinsically more judgement laden. Positive skews may be related to methodological problems such as poorly worded items [3].

#### Present state and recall bias

Retrospective items have been found to produce more favourable results than prospectively monitored health status data from the same patients [63,65]. This may be due to present state bias with retrospective judgements. However, retrospective questions that are least likely to be influenced by present state bias are transition questions [59].

#### The role of expectations

Different populations have different values, expectations and experiences, and these are likely to influence the results of patient surveys of satisfaction with treatment outcomes, or change in health status as a consequence of treatment. Particularly treatment satisfaction questions, as differing expectations have been shown to predict overall satisfaction [66], and expectations are likely to be influenced by a range of patient and socio-economic variables. Research on patient satisfaction attempts to overcome the effect of this by collecting baseline data and controlling for the influence of these factors [3]; however, in routine clinical settings this is unlikely to be possible.

#### Timing of survey

The point at which the measure is completed is likely to influence the response rate/responses provided - the consensus is that surveys should be conducted as soon after the medical encounter as possible to minimise recall bias [64,67,68]. There is evidence that HTQs used for clinical evaluative purposes in the short term (i.e. 12 weeks or less), may therefore be less likely to suffer from recall and non-response biases [40].

#### Question types and & response formats

There are two types of question routinely used to survey patients with regards to their hospital or treatment experiences; ratings and reports. The former are inherently more subjective and involve an evaluative element (i.e. are you satisfied with the outcome of your treatment?), and the latter are intentionally factual, more objective, and directly ask patients the extent to which their health has changed as a consequence of treatment (i.e. *did the treatment that you received improve health?*) [69]. Reports produce more variability and less skew, and are less likely to be associated with patient socio-demographic variables than ratings [64]. Therefore, questions that directly ask patients the extent to which an intervention has helped improve their health, or that probe their direct experience of treatment may be less subject to bias than questions that ask patients how satisfied they are with the outcomes of their treatment [69,70].

Responses to questions posed in surveys are generally categorical or numerical. Responses that range from “very satisfied” to “very unsatisfied” are the most commonly used rating style in satisfaction with treatment surveys. However, there are number of problems associated with these responses, for example a change from satisfied to dissatisfied may represent either an accumulation of small shifts in separate component areas or large shifts in a single component [67]. Furthermore, responses to this form of rating tend to fall into two narrow bands, being only superficially indicative of high satisfaction. Whilst a substantial change in ratings is usually obvious for certain interventions such hip replacement etc., a change in ratings may be less marked when small and subtle health status changes occur *i.e.* in cancer patients or patients with long term conditions.

Numerical and continuous scoring of satisfaction ratings using a Likert-type scales may provide a high degree of precision, but it should be remembered that actual values may be small and that these are not intervals [71]. This approach therefore risks providing a sense of pseudo-precision [71].

Responses can also be weighted according to their relative importance to the patient [67], however a problem with this method arises when the direct approach is used, as patients assign a numerical value of importance of equal numbers to each dimension [72]. The indirect approach requires the researchers to assign weightings by studying patient responses, but this method is flawed since it is difficult to extract information about how patients weigh and combine individually different dimensions to give their response [73]. The above problems bring into question the use of an amalgamated overall satisfaction score, namely that it provides over inflated results and masks areas of dissatisfaction with health care [30].

The response format used for the PJHS was that adopted from the Medical Outcome Study *i.e.* a 5-point rating from ‘excellent’ to ‘poor’ [74]. The authors used this format as it measured patient ratings of quality rather than satisfaction or other attitudes, they also reported that it performed well psychometrically and allowed for direct comparison between features of care [11]. The patient PGIC and many of the Oxford Measures described above use a similar approach (‘no change’ to ‘a great deal better’).

#### Patient differences

Patient factors have been shown to influence evaluations of hospital care. For example, evidence from a meta-analysis of socio-demographic factors and patient satisfaction surveys of medical care, that older, white, male patients, are more satisfied than other patients [75]. Greater self-perceived illness during in-patient stay has also been associated with poorer evaluations of care [76]. Post hospitalisation surveys may also under-represent males and older patients unless these groups are targeted by more vigorous follow-up methods [77].

### Methods to reduce bias

Methods to reduce bias in patient outcome surveys have been described as a) the use of qualitative methods in the design of the questionnaire, b) the use of patient reports rather than ratings, c) a balance of questions using both positively and negatively worded statements, and d) questions posed with better response scales [3]. Therefore the most important methodological consideration relates to the type of questionnaire used. One which contains open-ended questions will allow patients to specify and comment on the areas of care that they are referring to, whilst close ended questions gives a more quantitative evaluation. Ideally a survey should include both [67].

Preferably, satisfaction with, or the assessment of the outcomes of treatment should be measured multi-dimensionally [29]. This is to avoid the problems of using a single global measure which is likely to reflect numerous features of the treatment received, and be closely related to the quality of the care received [2]. In addition, multi-item scales generally yield more score variability, and higher reliability and validity scores than single items measures [78]. Ware et al. [79] also recommends self-administration of measures to reduce data collection costs and increase confidentiality, and placing questions about overall satisfaction or quality of care after all questions of a more specific nature.

Research on the reporting of health events has shown that the likelihood of its report is related to the importance of the event for the respondent [29]. Therefore the under-reporting of events of less significance may due to the fact that the information was not elicited by the data collection method. The reporting of such events has been improved by questionnaires that are designed to facilitate recall processes i.e. direct questions about a specific treatment process *(ibid)*.

## Conclusions

The findings of this review suggest that there are a number of benefits associated with combining a simple set of questions to elicit patients’ assessments of the outcomes of hospital treatment. Most importantly, asking patients to directly assess the impact of the treatment that they receive reflects outcomes relevant to the patient, which can often differ from clinical and provider appraisals of the same treatment or intervention. Directly asking patients to assess outcomes of treatment is also more likely to reflect patient report in routine clinical practice, as it is inherently natural for clinicians to ask patients questions in this way. The results of surveys using this type of question may therefore be more readily translated into clinical practice. There is also evidence in the literature that asking patients to assess the outcome of their treatment has face validity. This is shown in the widespread use of such questions, particularly transition questions as external anchors to assess the performance of other PROMs. Furthermore, a number of well-developed disease specific and generic measures using the same question format have been substantially validated, and therefore provide some evidence of construct validity. The advantages of using a simple set questions that ask patients to assess outcomes of treatment they receive also appear to be borne out it in terms of how much time and resources they save in comparison to administering and analysing lengthy measures.

There are also a number of problems associated with the above approaches that can result in positive data skews. In balance however, it would appear that these problems are associated with PRO surveys in general, and there are strategies that can minimise these risks. Firstly, the use of reports rather than ratings may help increase variability in responses and minimise positive skews. Furthermore, combining a simple transition question as a temporal marker or probed recall aid, alongside a retrospective global report of the outcomes of treatment may overcome some of the problems associated with recall and present state bias. The inclusion of a question which probes the negative aspects of treatment such as unexpected events or side effects will ensure that all features of experience are covered. Timing the administration of the questionnaire within the first three months post intervention may also reduce non-response bias, and minimise recall bias, and ensure that patients less likely to respond (i.e. men) are prudently followed-up, this may produce a more representative data set.

The findings from this review suggest that a simple set of items that directly ask patients to assess the outcome of their treatment could be tested for use as an evaluative measure for generic use in a range of settings.

## Competing interests

The authors declare that they have no competing interests.

## Authors’ contributions

HL conducted the review and wrote the report and subsequent article for publication. CJ, RF, MH and EG were part of the research team and commented on drafts of the manuscript. All authors read and approved the final manuscript.
